# Twenty‐Year Outcome and Association Between Early Treatment and Mortality and Disability in an Inception Cohort of Patients With Rheumatoid Arthritis: Results From the Norfolk Arthritis Register

**DOI:** 10.1002/art.40090

**Published:** 2017-07-10

**Authors:** James M. Gwinnutt, Deborah P. M. Symmons, Alexander J. MacGregor, Jacqueline R. Chipping, Tarnya Marshall, Mark Lunt, Suzanne M. M. Verstappen

**Affiliations:** ^1^ Arthritis Research UK Centre for Epidemiology, University of Manchester Manchester UK; ^2^ Arthritis Research UK Centre for Epidemiology, University of Manchester and Central Manchester University Hospitals NHS Foundation Trust Manchester UK; ^3^ Norfolk and Norwich University Hospitals NHS Trust and University of East Anglia Norwich UK

## Abstract

**Objective:**

To describe the outcome in patients with rheumatoid arthritis (RA) over 20 years from symptom onset, and to assess the association between early treatment (with disease‐modifying antirheumatic drugs/steroids) and mortality and disability during follow‐up.

**Methods:**

Patients recruited to the Norfolk Arthritis Register (NOAR) between 1990 and 1994 who met the 2010 American College of Rheumatology/European League Against Rheumatism RA criteria at baseline were included in this analysis. Demographic and clinical variables were collected at baseline and at years 1–3, 5, 7, 10, 15, and 20. Disease activity (swollen joint count [SJC]/tender joint count [TJC]), disability (Health Assessment Questionnaire disability index [HAQ DI]), and mortality over 20 years were determined. Associations between treatment group (early treatment [ET], treatment ≤6 months after symptom onset; late treatment [LT], treatment >6 months after symptom onset; never treatment [NT], no treatment) and mortality and disability were assessed using weighted pooled logistic regression and weighted multilevel mixed‐effects linear regression, respectively. Inverse weights were used to account for confounding by indication and censoring.

**Results:**

This study included 602 patients with RA (median age 56 years [interquartile range 44–68 years]; 65.9% women). The median SJCs and TJCs were low during the follow‐up period (1–3 swollen joints and 3–6 tender joints). The median HAQ DI score increased after year 1 but remained at low/moderate levels (median 1.25 after year 10). The risk of mortality was reduced in the ET and LT groups compared with that in the NT group. The ET group and the NT group had comparable HAQ DI scores during the follow‐up period (β = 0.03, 95% confidence interval [95% CI] −0.06, 0.12), while the HAQ DI score was increased in the LT group (for LT versus NT, β = 0.10 [95% CI 0.02, 0.17]).

**Conclusion:**

The results of this study indicate the importance of early treatment with regard to the long‐term outcomes in patients with RA.

Early rheumatoid arthritis (RA), a type of inflammatory polyarthritis, is a chronic disease primarily characterized by synovial joint inflammation 1. Both conditions (RA and inflammatory polyarthritis) may lead to progressive joint destruction and premature mortality 2, 3, 4, 5. With appropriate therapy, however, this outcome can potentially be ameliorated 6, 7, 8, although there are relatively few studies investigating the long‐term outcome in patients with RA 9, 10, 11, 12, 13, 14, 15, 16. A previous study from the UK described the outcomes in 112 patients with RA who were recruited between 1964 and 1966 (prevalent cases) (mean age at symptom onset 45 years). Twenty years later, 35% of these patients had died, and an additional 43% of the patients were classified as having moderate‐to‐high functional disability 17. The patients in this cohort were predominantly treated with steroids, gold, and chloroquine, which were standard treatments at that time.

Gathering new data on outcomes in the modern era is important, because over the last 2 decades there have been significant advances in available treatments and strategies for the management of RA 18, 19. Methotrexate (MTX) has become the first‐choice synthetic disease‐modifying antirheumatic drug (DMARD) 18, and a number of biologic DMARDs have been introduced since 2000 20. Previous research in patients with RA demonstrated an association between a reduced mortality risk and ever‐exposure to MTX treatment over a mean of 6 years 6, reduced mortality risk and exposure to tumor necrosis factor inhibitor (TNFi) treatment over a mean of 4.9 years 21, as well as improved functional disability with MTX and TNFi therapy 22, 23, 24, 25.

The aforementioned advances in treatment strategy included a shift toward initiating synthetic DMARD therapy early in the disease course, with the aim of treating patients within a “window of opportunity” in order to achieve maximal beneficial outcome 26, 27. We previously showed that in patients with early inflammatory arthritis who received early treatment with synthetic DMARDs or steroids (initiation <6 months after symptom onset), the odds of having a high score (≥1) on the Health Assessment Questionnaire disability index (HAQ DI) 28 at 5 years were comparable with the odds in patients who never required treatment, after adjustment for differences in disease severity between the treatment groups. Those in whom therapy was initiated later (≥6 months after symptom onset) had 2‐fold increased odds of having high HAQ DI scores compared with those who were never treated 29. Furthermore, those treated early showed a decrease in HAQ DI scores from baseline to year 10, which approached significance, while those treated later had increased HAQ DI scores at year 10 compared with baseline, again after adjustment for differences in disease severity between groups 30. Other groups of investigators have also shown the negative consequences of delayed assessment in terms of remission rates and joint destruction 31. However, the benefits of early treatment have yet to be demonstrated to extend past 10 years. It is also possible that the underlying natural history of RA may have changed over time 32.

In the current study, we investigated the outcome in patients with early RA over the course of 20 years, including mortality, disease activity, and physical function. We also examined the relationship between early synthetic DMARD or steroid therapy and mortality risk and physical function over 20 years.

## PATIENTS AND METHODS

A total of 1,098 patients with inflammatory polyarthritis were recruited by the Norfolk Arthritis Register (NOAR) between 1990 and 1994. Detailed information about NOAR is presented elsewhere 33. Briefly, NOAR aimed to recruit and follow up prospectively all incident cases of inflammatory polyarthritis in the former Norwich Health Authority region, Norfolk, UK. These patients with inflammatory polyarthritis were referred by primary care physicians (a large majority of the UK population [99%] is registered with a primary care practice) or consultant rheumatologists. The inclusion criteria for this analysis were as follows: ≥16 years of age and ≥2 swollen joints for ≥4 weeks. Patients were excluded if they were recruited more than 2 years following symptom onset (76 patients were excluded). For this analysis, only patients who met the American College of Rheumatology (ACR)/European League Against Rheumatism (EULAR) 2010 criteria for RA were included 34. The criteria were applied retrospectively to the baseline characteristics of the patients who were included (n = 614). Patients gave written consent, and the study was approved by the Norfolk and Norwich University Hospital Local Research Ethics Committee.

### Assessments

Patients were assessed at baseline and at 1, 2, 3, 5, 7, 10, 15, and 20 years after registration. Patients were assessed beyond year 5 only if they had documented swollen joints on ≥2 occasions or had received DMARDs or oral corticosteroids by the fifth‐year assessment (8 patients were excluded). Date of birth and sex were recorded at baseline. At each assessment, research nurses administered a standardized questionnaire (including smoking status [never/former/current]). The start and stop dates of treatment with all DMARDs and steroids were collected from patients at each follow‐up visit. Patients who were receiving synthetic DMARDs or steroids before symptom onset (n = 12) were excluded from the analysis. Patients were classified into 1 of 3 treatment groups, with treatment being synthetic DMARDs and steroids: the early treatment (ET) group, in which patients received treatment ≤6 months after symptom onset; the late treatment (LT) group, in which patients received treatment >6 months after onset; and the never treatment (NT) group, in which patients never received treatment.

Comorbidities were self‐reported at and after baseline and were coded based on the relevant chapters of the International Classification of Diseases, Ninth Edition and Tenth Edition (see Supplementary File 1, available on the *Arthritis & Rheumatology* web site at http://onlinelibrary.wiley.com/doi/10.1002/art.40090/abstract). Once a patient reported a comorbidity, he or she was coded as having that comorbidity throughout the remainder of the follow‐up period. Research nurses performed swollen joint counts (SJCs) and tender joint counts (TJCs) in 51 joints, from which 28‐joint counts were derived (except at follow‐up visits 5 and 7). Blood samples were obtained at baseline. Serum was stored frozen, for determination of C‐reactive protein (CRP; mg/liter), rheumatoid factor (RF; latex agglutination test, positive cut‐off 40 units/ml), and anti–cyclic citrullinated peptide 2 (anti–CCP‐2) antibodies (tested using a Diastat Anti‐CCP kit, cutoff 5 units/ml [Axis‐Shield]). Blood samples were then obtained every 5 years; in these samples, only the CRP level was determined. Disease activity was measured with the Disease Activity Score, using 3 variables: the number of swollen joints, the number of tender joints, and the CRP level 35, when CRP data were available.

### Outcomes

Disease activity was analyzed based on the SJC in 51 joints and the TJC in 51 joints, as described above. All patients were flagged with the Office for National Statistics (ONS), the holders of the UK death register, which provided copies of death certificates including date of death and the underlying cause of death. The ONS also provided age‐ and sex‐specific mortality rates by calendar year for the Norfolk population (1990–2013). Patients were censored at their 20th anniversary assessment or 20 years after symptom onset (if they did not attend the 20th assessment), with the exception of patients who left the country, who were censored on their departure date (7 patients with RA). At each assessment, patients completed the British Modification of the HAQ disability index 36, a validated self‐report measure of functional disability that yields a score from 0 (no disability) to 3 (maximum disability).

### Statistical analysis

Descriptive statistics were used to describe baseline demographic and clinical variables. Baseline scores were compared between treatment groups using the Kruskal‐Wallis or chi‐square test, depending on the type and distribution of data.

The standardized mortality ratio (SMR) was calculated for the cohort by comparing the observed numbers of deaths with the expected number of deaths for the local population based on age‐ and sex‐specific mortality rates. Patients were included in this analysis until death, 20 years after symptom onset, or the end of 2013, whichever came first. The association between baseline variables, including age, sex, SJC in 51 joints, TJC in 51 joints, HAQ DI, CRP, RF, and anti–CCP‐2, and mortality was assessed using a multivariate Cox proportional hazards model. To assess the association between treatment group and mortality, initially a Cox proportional hazards model, adjusted for age and sex, was used, and Kaplan‐Meier survival curves were plotted. The SMR for each treatment group was also calculated. To account for confounding by indication, a weighted multivariate pooled logistic regression model was used 37, 38, which produces odds ratios (ORs) that are equivalent to hazard ratios. Covariates included baseline measures (sex, smoking status, RF, anti–CCP‐2, HAQ DI, 51‐joint SJC, 51‐joint TJC, and CRP), and data for time‐varying measures where collected (HAQ DI, 51‐joint SJC, 51‐joint TJC, CRP, and comorbidities).

The median 51‐joint SJC, 51‐joint TJC, and HAQ DI scores and the numbers and proportions of patients receiving synthetic DMARDs were determined for each time point. The relationship between treatment group and disability over 20 years was assessed using a multilevel mixed‐effects linear regression model, which is a longitudinal regression model that allows time‐varying weights. Initially, only age and sex were controlled for using an unweighted model. To account for confounding by indication and censoring, a weighted multivariate model was then used, with the same covariates as those used for the pooled logistic regression described above (except time‐varying HAQ DI scores).

To control for confounding by indication and censoring, inverse probability weights, which account for cumulative treatment exposure as well as censoring, were used to weight the covariates in the regression models (see Supplementary File 2, available on the *Arthritis & Rheumatology* web site at http://onlinelibrary.wiley.com/doi/10.1002/art.40090/abstract), using methods adapted from those described by Fewell et al 37. Multiple imputation, using iterative chained equations (5 imputed data sets were created) conditioned on baseline, lagged, and current assessment variables, was used to account for missing data on the assessments that patients attended. All regression analyses were also performed in the total population of patients with inflammatory polyarthritis (n = 1,000). Analysis was performed using Stata statistical software version 13.1 (StataCorp; 2013).

## RESULTS

A total of 602 patients with RA were recruited between 1990 and 1994 and met all inclusion/exclusion criteria (median symptom duration 5.4 months, interquartile range [IQR] 2.9–9.9). The median age at symptom onset was 56 years (IQR 44–68 years), and 397 patients (65.9%) were women. The baseline characteristics of the patients are shown in Table 1.

**Table 1:** Baseline characteristics of the total cohort of RA patients and according to treatment group*

Characteristic	Cohort (n = 602)	ET (n =160)	LT (n = 249)	NT (n =193)	*P* †
Age at onset, years	56 (44–68)	62 (49–71)	54 (44–65)	55 (42–67)	0.0006‡
Female sex, no. (%)	397 (66)	85 (53.1)	180 (72.3)	132 (68.4)	<0.001§
Symptom duration, months	5.4 (2.9–9.9)	2.7 (2.4–5.9)	7.4 (3.8–12.0)	5.1 (2.7–9.5)	0.0001‡
SJC in 28 joints	9 (4–14)	10 (5–16)	9 (4–14)	8 (4–13)	0.0243‡
SJC in 51 joints	11 (6–17)	13 (6.5–20)	11 (6–18)	10 (5–15)	0.0247‡
TJC in 28 joints	9.5 (4–16)	10 (4–17)	8 (4–15)	10 (6–16)	0.1503‡
TJC in 51 joints	14 (7–23)	13 (5–22.5)	13 (6–23)	15 (9–22)	0.2272‡
CRP, mg/liter¶	8 (1–22)	13.5 (6.8–38)	8 (2–19)	3 (0–11)	0.0001‡
DAS28#	4.6 (3.8–5.5)	5.1 (4.1–6.0)	4.5 (3.8–5.5)	4.4 (3.7–5.3)	0.0011‡
HAQ DI score**	1.00 (0.50–1.63)	1.25 (0.63–1.88)	1.00 (0.50–1.63)	0.88 (0.25–1.50)	0.0001‡
Smoking history, no. (%)					
Never smoker	177 (29.4)	42 (26.3)	80 (32.1)	55 (28.5)	0.441§
Former smoker	255 (42.4)	76 (47.5)	95 (38.2)	84 (43.5)	
Current smoker	170 (28.2)	42 (26.3)	74 (29.7)	54 (28.0)	
RF status††					
Positive, no. (%)	221 (40.3)	71 (46.4)	112 (49.8)	38 (22.4)	<0.001§
Negative, no. (%)	327 (59.7)	82 (53.6)	113 (50.2)	132 (77.7)	
Anti‐CCP status§§					
Positive, no. (%)	202 (41.1)	73 (52.5)	106 (54.6)	23 (14.6)	<0.001§
Negative, no. (%)	289 (58.9)	66 (47.5)	88 (45.4)	135 (85.4)	
Current synthetic DMARD treatment, no. (%)	109 (18.1)	75 (46.9)	34 (13.7)	0 (0.0)	<0.001§
Time to first treatment, months	8.4 (4.0–19.1)	3.1 (2.0–5.0)	15.6 (10.0–36.2)	–	<0.0001‡

* Complete data (100%) were available for all variables in all treatment groups, except for C‐reactive protein (CRP), Disease Activity Score in 28 joints (DAS28), Health Assessment Questionnaire disability index (HAQ DI), rheumatoid factor (RF), and anti–cyclic citrullinated peptide (anti‐CCP). Except where indicated otherwise, values are the median (interquartile range). RA = rheumatoid arthritis; SJC = swollen joint count; TJC = tender joint count.

† Across treatment groups.

‡ By Kruskal‐Wallis test.

§ By chi‐square test.

¶ The percent complete data for CRP level was 84.2% in the total cohort, 88.8% in the early treatment (ET) group (treatment ≤6 months after symptom onset), 83.1% in the late‐treatment (LT) group (treatment >6 months after symptom onset), and 81.9% in the never treatment (NT) group (patient never received disease‐modifying antirheumatic drugs [DMARDs] or steroids during follow‐up).

# The percent complete data was 99.9% in the total cohort, 98.8% in the ET group, 98.8% in the LT group, and 99.5% in the NT group.

** The percent complete data was 99.9% in the total cohort, 98.8% in the ET group, 98.8% in the LT group, and 99.5% in the NT group.

†† The percent complete data was 91.0% in the total cohort, 95.6% in the ET group, 90.3% in the LT group, and 88.1% in the NT group.

§§ The percent complete data was 81.6% in the total cohort, 86.9% in the ET group, 77.9% in the LT group, and 88.1% in the NT group.

In total, 160 patients (26.6%) received treatment within 6 months of symptom onset. Among these patients, 94 (58.8%) were prescribed sulfasalazine (SSZ), 45 (28.1%) were prescribed steroids, 8 (5.0%) were prescribed MTX, and 13 (8.1%) were prescribed other DMARDs. Among the remaining 442 patients, 88 (19.9%) received their first treatment within 6–12 months, 77 (17.4%) received treatment at 1–2 years, 84 (19.0%) received treatment ≥2 years after symptom onset, and 193 (43.7%) never received treatment while attending follow‐up. Male patients were more likely to receive early treatment (n = 75 [46.9%]) compared with late treatment (n = 69 [27.7%]), and patients who received early treatment had a shorter disease duration at presentation (median 2.7 months) compared with those who received late treatment (median 7.4 months). Patients who received early treatment had worse clinical characteristics for all baseline variables compared with those who received late treatment or no treatment, except for TJCs and autoantibody status (Table 1). Similar results were seen when all patients with inflammatory polyarthritis were included (for details, see Supplementary File 3, available on the *Arthritis & Rheumatology* web site at http://onlinelibrary.wiley.com/doi/10.1002/art.40090/abstract).

A total of 207 patients (34.4%) were available for the 20‐year assessment. During the follow‐up period, 205 patients (34.1%) left the cohort due to death, 135 (22.4%) declined further follow‐up visits, 8 (1.3%) became ineligible at year 5, and 47 (7.8%) were lost to follow‐up (for details, see Supplementary File 4, available on the *Arthritis & Rheumatology* web site at http://onlinelibrary.wiley.com/doi/10.1002/art.40090/abstract).

Because all patients were flagged with the ONS, mortality data were complete for the whole cohort up to the 20th anniversary assessment (even if follow‐up ceased), except those who left the country. During 9,774 person‐years of follow‐up (mean 14.8 years), 265 patients (44.0%) died. The age‐ and sex‐standardized SMR for the cohort of patients with RA was increased compared with that for the general population in the Norfolk area (SMR 1.25 [95% CI 1.11, 1.42]). In a multivariate Cox regression analysis, older age at symptom onset (hazard ratio [HR] 1.10 per increased year of age at onset [95% CI 1.08, 1.11]) and male sex (HR 1.47 [95% CI 1.13, 1.92]) were associated with increased risk of death during that time period. Being a current smoker at baseline was independently associated with an increased risk of death compared with never smokers (HR 1.65 [95% CI 1.17, 2.33]) and former smokers (HR 1.82 [95% CI 1.34, 2.45]).

**Figure 1:**
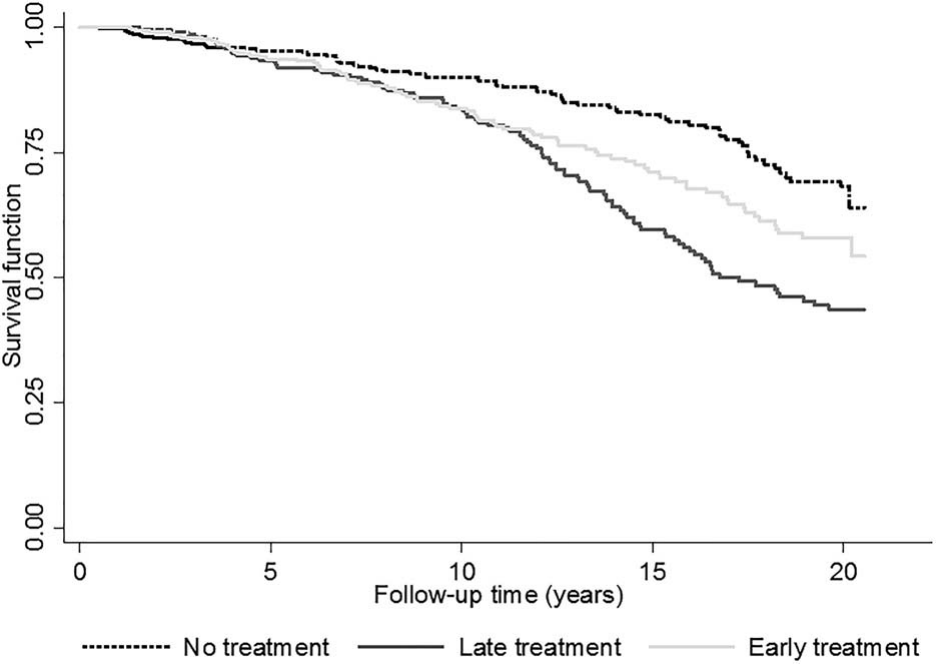
Survival curves for rheumatoid arthritis patients in the 3 treatment groups, after adjustment for age and sex.

In total, 88 (55.0%) of the patients who received early treatment died over the course of follow‐up compared with 99 (39.8%) of the patients who received late treatment and 78 (40.4%) of those who received no treatment. The SMR was increased in the ET group (SMR 1.28 [95% CI 1.03, 1.59]) and the LT group (SMR 1.23 [95% CI 1.01, 1.51]), and there was a trend toward significance for the NT group (SMR 1.25 [95% CI 0.99, 1.57]) compared with the general population of Norfolk. Within the cohort, there was little difference in the risk of death between treatment groups, after adjustment for age and sex (ET versus NT, HR 1.09 [95% CI 0.80, 1.11]; LT versus NT, HR 0.99 [95% CI 0.73, 1.33]) (Figure 1). After weighting to account for confounding by indication, the association between time to first treatment and mortality risk remained nonsignificant for the ET group, although there was a trend toward reduced risk of mortality (ET versus NT, adjusted OR [OR_adj_] 0.78 [95% CI 0.54, 1.11]; LT versus NT, OR_adj_ 0.66 [95% CI 0.47, 0.92]). When all patients with inflammatory polyarthritis were included (n = 1,000), the results were similar (ET versus NT, OR_adj_ 0.82 [95% CI 0.61, 1.11]; LT versus NT, OR_adj_ 0.78 [95% CI 0.59, 1.03]).

**Figure 2:**
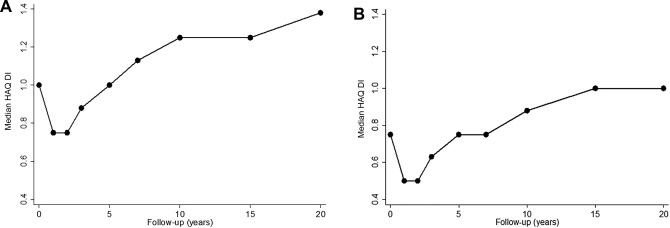
Median Health Assessment Questionnaire disability index (HAQ DI) score at each follow‐up visit in patients with rheumatoid arthritis (**A**) and the full cohort of patients with inflammatory polyarthritis (**B**).

Table 2 shows the median disease activity and functional disability scores over follow‐up for the patients with RA, stratified by treatment group. All patients were included in the analysis up to the point of their last follow‐up visit. The median SJCs and TJCs decreased rapidly in the first year after baseline (median 11 swollen joints and 14 tender joints at baseline) and remained low throughout follow‐up (median SJC range 1–3, and median TJC range 3–6). Median HAQ DI scores for the total RA cohort also declined initially but then increased from the year 2 assessment onward, culminating in a median score higher than the baseline score after year 7 (median HAQ DI score of 1.25 after year 10 [IQR 0.50–2.00]) (Figure 2). The proportion of patients receiving synthetic DMARDs was higher in the ET group compared with the LT group until assessment 2, after which time the proportions were comparable (55–65%) (Table 2). Only a small proportion of patients received biologic DMARDs during the study (7.6%), and these patients were evenly distributed between the ET group and the LT group (19 [11.9%] of 160 patients in the ET group, and 27 [10.8%] of 249 patients in the LT group). Similar trends were observed when all patients with inflammatory polyarthritis were included, although with lower median scores due to lower disease severity in this group (see Supplementary File 3, available on the *Arthritis & Rheumatology* web site at http://onlinelibrary.wiley.com/doi/10.1002/art.40090/abstract).

**Table 2:** SJC and TJC in 51 joints, HAQ DI scores, and proportion of the 602 RA patients receiving synthetic DMARDs over 20‐year follow‐up, stratified by treatment group^a^

	Follow‐up year								
	0	1	2	3	5	7	10	15	20
SJC in 51 joints									
Total cohort	11 (6–17)	3 (1–9)	2 (0–8)	2 (0–7)	–	–	2 (0–5)	1 (0–4)	1 (0–3)
ET	13 (6.5–20)	4 (1–10)	3 (0–8)	2.5 (0–9)	–	–	2 (0–6)	1 (0–4)	0.5 (0–2)
LT	11 (6–18)	4 (1–11)	3 (1–9)	3 (0–9)	–	–	2 (0–7)	1 (0–5)	1 (0–4)
NT	10 (5–15)	2 (1–8)	1 (0–6)	2 (0–5)	–	–	1 (0–3)	0.5 (0–2)	0 (0–2)
TJC in 51 joints									
Total cohort	14 (7–23)	6 (2–15)	5 (1–15)	5 (1–15)	–	–	3 (0–12)	4 (0–15)	4 (0–12)
ET	13 (5–22.5)	5 (1–15)	4 (1–13)	4.5 (0–14.5)	–	–	2 (0–11)	2 (0–12)	2.5 (0–5)
LT	13 (6–23)	7 (1–17)	7 (2–18)	5 (2–16.5)	–	–	4 (1–15)	6 (1–19.5)	6 (1–16)
NT	15 (9–22)	6 (2–14)	4 (1–12)	4 (1–10.5)	–	–	3 (0–10)	3.5 (1–12)	4 (0–7)
HAQ DI score									
Total cohort	1.00 (0.50–1.63)	0.75 (0.25–1.50)	0.75 (0.25–1.63)	0.88 (0.25–1.63)	1.00 (0.38–1.75)	1.13 (0.50–1.88)	1.25 (0.38–2.00)	1.25 (0.50–2.00)	1.38 (0.50–2.00)
ET	1.25 (0.63–1.88)	0.75 (0.13–1.56)	0.88 (0.25–1.75)	0.88 (0.25–1.88)	1.06 (0.38–1.88)	1.13 (0.50–1.88)	1.25 (0.38–2.13)	1.38 (0.75–2.13)	1.38 (0.81–2.00)
LT	1.00 (0.50–1.63)	0.88 (0.38–1.63)	1.00 (0.25–1.75)	1.13 (0.38–1.75)	1.13 (0.50–1.88)	1.25 (0.50–1.88)	1.50 (0.63–2.00)	1.50 (0.63–2.00)	1.50 (0.69–2.13)
NT	0.88 (0.25–1.50)	0.50 (0.13–1.25)	0.50 (0.13–1.13)	0.63 (0.00–1.25)	0.88 (0.25–1.50)	0.88 (0.25–1.50)	0.88 (0.25–1.38)	1.00 (0.25–1.75)	0.88 (0.25–1.63)
Synthetic DMARD, no. (%)									
Total cohort	109 (18.1)	204 (35.7)	203 (38.5)	207 (41.0)	179 (38.3)	160 (40.6)	153 (40.9)	127 (45.2)	98 (47.3)
ET	75 (46.9)	98 (62.4)	93 (63.7)	85 (59.4)	69 (45.8)	56 (53.9)	55 (57.3)	45 (60.0)	31 (57.4)
LT	34 (13.7)	106 (43.1)	110 (46.4)	122 (54.0)	110 (52.1)	104 (55.6)	98 (54.8)	82 (58.6)	67 (62.0)
NT	0 (0.0)	0 (0.0)	0 (0.0)	0 (0.0)	0 (0.0)	0 (0.0)	0 (0.0)	0 (0.0)	0 (0.0)

a Except where indicated otherwise, values are the median (interquartile range). SJC = swollen joint count; TJC = tender joint count; HAQ DI = Health Assessment Questionnaire disability index; RA = rheumatoid arthritis; ET = early treatment (treatment ≤6 months after symptom onset); LT = late treatment (treatment >6 months after symptom onset); NT = never treatment (patient never received disease‐modifying antirheumatic drugs [DMARDs] or steroids during follow‐up).

Swollen and tender joint counts were similar between treatment groups over the follow‐up period, excluding the baseline assessment. However, functional disability was consistently higher in patients who received treatment compared with patients who did not (Table 2).

After controlling for age and sex, there was an association between being prescribed treatment and increased HAQ DI scores over follow‐up (Table 3). When the model was fully adjusted and inverse probability weights were applied to account for confounding by indication, patients in the ET group had HAQ DI scores during follow‐up similar to those in the NT group (for ET versus NT, β = 0.03 [95% CI −0.06, 0.12]), whereas those in the LT group had significantly higher disability scores over follow‐up (LT versus NT, β = 0.10 [95% CI 0.02, 0.17]). Similar results were observed when all patients with inflammatory polyarthritis were included in the analysis.

**Table 3:** Association between treatment regimen and the HAQ DI score over 20 years^a^

	RA patients		Total cohort	
Model, treatment regimen	No. of patients	β (95% CI)	No. of patients	β (95% CI)
Adjusted for age and sex				
NT	193	0	442	0
LT	249	0.27 (0.15, 0.39)	347	0.37 (0.28, 0.46)
ET	160	0.25 (0.11, 0.38)	211	0.36 (0.26, 0.47)
Fully adjusted^b^				
NT	193	0	442	0
LT	249	0.10 (0.02, 0.17)	347	0.11 (0.06, 0.17)
ET	160	0.03 (–0.06, 0.12)	211	0.04 (–0.03, 0.11)

a RA = rheumatoid arthritis; ET = early treatment (treatment ≤6 months after symptom onset); LT = late treatment (treatment >6 months after symptom onset); NT = never treatment (patient never received disease‐modifying antirheumatic drugs or steroids during follow‐up).

b Adjusted for sex and baseline anti–citrullinated protein antibodies, rheumatoid factor, smoking status, Health Assessment Questionnare disability index (HAQ DI), swollen joint count (SJC), tender joint count (TJC), and C‐reactive protein (CRP) level, and for time‐varying measures, including age, CRP level, SJC, TJC, and comorbidities, and weighted using inverse probability of treatment/censoring weights.

## DISCUSSION

This study has 2 important messages: the first concerns the long‐term outcome in patients with RA in the modern era treated according to best practice at the time of presentation, and the second pertains to the benefit of early treatment, which is still apparent into the second decade after symptom onset with respect to functional disability.

Median SJCs and TJCs decreased after baseline and remained low during the follow‐up period. Median functional disability decreased initially but then increased over time to above baseline levels by year 7 and then continued to increase up to year 20. Kapetanovic et al followed 183 Swedish patients with early RA for 20 years and showed a similar HAQ DI trajectory, with mean HAQ DI scores increasing to >1.0 at year 10 and thereafter 13. This level of functional disability is higher than that reported in healthy individuals of a similar age. In a random sample of adults in central Finland, the mean HAQ DI scores for all groups of individuals younger than age 75 years were <0.5 39. Similar results were observed in a random sample of individuals older than age 65 years in Augsburg and Aichach‐Friedberg, Germany (only 22.5% of the cohort had an HAQ DI score of ≥0.5) 40.

Despite the increase in median HAQ DI scores over 20 years, these results are encouraging. An HAQ DI score of 1.38 represents low to moderate disability 41. In contrast, a study that recruited 112 patients with RA in Droitwich, UK from 1964 to 1966 and followed them for a similar time period showed much higher levels of disability at 20 years 17. It thus appears that long‐term disability in RA patients is now less severe. Furthermore, although the proportion of patients who died was similar in the Droitwich and NOAR studies, patients in the NOAR cohort had an older mean age at symptom onset (56 years versus 45 years in the Droitwich cohort).

The second finding from this study relates to the benefits of early DMARD therapy. We previously showed that treatment within the first 6 months following symptom onset is associated with benefits in physical function at 5 and 10 years, after appropriate adjustment for differences in disease severity 29, 30. In the current analysis, we observed that, after adjustment for confounding by indication (i.e., adjusting for demographic and disease factors that may have influenced the decision to start DMARD or steroid treatment at baseline or subsequently), patients who were treated early had levels of disability over the follow‐up period that were similar to those in patients who did not receive treatment, while patients treated later had significantly higher levels of disability over 20 years. This supports the importance of the “window of opportunity” construct for treatment, showing that early treatment leads to improved outcomes even into the second decade following symptom onset. Increased functional disability over time could be attributable to worse joint damage 42, 43, and it has been shown that patients who receive later treatment have higher radiologic scores at follow‐up compared with those treated early 8, 44, 45.

The SMRs for the total population and for the 2 treatment groups were elevated. The SMR for those who were never treated was increased to a similar extent but failed to reach significance. There was a trend toward reduced mortality risk in the ET group and a significant reduction in mortality risk in the LT group compared with the NT group. Treatment with MTX has previously been shown to be associated with a reduced medium‐term risk of mortality in RA patients 6, and we previously showed that having disease in remission at least once within the first 3 years of follow‐up was associated with reduced mortality risk in patients with inflammatory polyarthritis compared with the risk in patients who never achieved remission within the first 3 years of follow‐up (median of 7.9 years) 46. It is the nature of the SMR that it approaches 1.00 as the length of follow‐up increases, because all patients will eventually die.

This analysis has a number of strengths. It is one of the largest inception cohorts of patients with RA and inflammatory polyarthritis with a 20‐year follow‐up period. By retrospectively applying the 2010 ACR/EULAR criteria for RA 34, we have been able to study the subgroup of patients classified as having RA according to the latest criteria and compare their results with those of patients in the whole NOAR cohort and other published RA cohorts. The use of inverse probability weights to a longitudinal regression model allows us to control for confounding by indication over time. This is the current state‐of‐the art approach in terms of controlling for confounding by indication and differs from approaches used in our previous work 28, 29.

The attrition rates in this cohort are to be expected over a 20‐year follow‐up period. The use of inverse probability of censoring weights minimizes attrition bias. Because SJCs and TJCs were not determined at follow‐up visits 5 and 7, these time points could not be included in the longitudinal analyses. However, because we assessed a majority of these patients at assessments 3 and 10, patient disability should be modeled sufficiently.

A surprisingly high proportion of patients (32.1%) never received DMARDs or steroids. This reflects the fact that patients were recruited from primary and secondary care settings and followed up regardless of disease severity. If the study had been entirely based on continuing follow‐up in secondary care, then patients with mild disease would have been discharged and contributed no follow‐up information. Our study shows that patients who never received DMARD treatment did well and served as a useful group with which to compare the groups of patients who were treated either early or late.

Finally, these patients were recruited more than 20 years ago, meaning that the standard treatment at that time does not reflect best treatment practices today. Although this is a limitation, treatment practices are constantly evolving. If we wish to understand the 20‐year outcome in RA patients, the only method is to study patients who were exposed to the treatment strategies used 20 years ago. Nevertheless, these results are still important, because they show clear improvement in these patients compared with patients studied in the prior 20‐year period and also show that early treatment with predominantly SSZ or steroids is associated with improved outcomes 20 years later. Thus, because MTX is considered a more effective treatment than SSZ, the benefits of early treatment with MTX may be even greater after 20 years.

In conclusion, this cohort of RA patients had relatively low median levels of disease activity from year 1 onward. In contrast, the median level of disability increased to above baseline levels after 7 years. This disability is still moderate. Thus, on average, patients experienced relatively good long‐term outcomes, particularly compared with those observed in an earlier UK cohort recruited from 1964 to 1966 and followed up for 20 years. As might be expected, early therapy was given to patients with more severe disease. After adjustment for this confounding by indication over time, functional disability was greater in the LT group compared with the NT group over the course of follow‐up, while the ET group had levels of disability similar to those in the NT group, indicating that the benefits of early treatment were sustained long term.

## AUTHOR CONTRIBUTIONS

All authors were involved in drafting the article or revising it critically for important intellectual content, and all authors approved the final version to be published. Dr. Verstappen had full access to all of the data in the study and takes responsibility for the integrity of the data and the accuracy of the data analysis.

### Study conception and design

Gwinnutt, Symmons, MacGregor, Verstappen.

### Acquisition of data

Chipping, Marshall.

### Analysis and interpretation of data

Gwinnutt, Symmons, Lunt, Verstappen.

## Supporting information

Supplementary file 1 – ICD9 and ICD10 codes used to classify comorbidities and comorbidities over time

Supplementary file 2

Supplementary file 3 – Baseline and follow‐up characteristics of the total IP population

Supplementary file 4 – attrition from the cohort over 20 years
