# Supplementary material for: Twenty‐Year Outcome and Association Between Early Treatment and Mortality and Disability in an Inception Cohort of Patients With Rheumatoid Arthritis: Results From the Norfolk Arthritis Register
Source: Arthritis Rheumatol. 2017 Jul 10;69(8):1566–75. doi: 10.1002/art.40090 (PMC5600136; doi:10.1002/art.40090)
Supplement: Supplementary file 1 — – ICD9 and ICD10 codes used to classify comorbidities and comorbidities over time [file ART-69-1566-s001.docx]

**Supplementary file 1 – ICD9 and ICD10 codes used to classify comorbidities and comorbidities over time**

| Comorbidity | ICD9 codes | ICD10 codes |
| --- | --- | --- |
| Neoplasms (chapter II) | 140 - 239 | C00 – D48 |
| Diseases of blood and blood forming organs and certain disorders involving the immune mechanism (chapter III) | 280 - 289 | D50 – D89 |
| Endocrine, nutritional and metabolic diseases (chapter IV) | 240 - 279 | E00 – E90 |
| Mental and behavioural disorders (chapter V) | 290 - 319 | F00 – F99 |
| Diseases of the nervous system (chapter VI) | 320 - 359 | G00 – G99 |
| Diseases of the circulatory system (chapter IX) | 390 - 459 | I00 – I99 |
| Diseases of the respiratory system (chapter X) | 460 - 519 | J00 – J99 |
| Diseases of the digestive system (chapter XI) | 520 - 579 | K00 – K93 |
| Diseases of the skin and subcutaneous tissues (chapter XII) | 680 - 709 | L00 – L99 |
| Diseases of the musculoskeletal system and connective tissue (chapter XIII) | 710 - 739 | M00 – M99 |

*Table 1 - The ICD 9 and ICD10 codes included when coding comorbidity variables*

| Comorbidity  *Table 1 cont. - The ICD 9 and ICD10 codes included when coding comorbidity variables* | ICD9 codes | ICD10 codes |
| --- | --- | --- |
| Angina | 413 413.0 413.1 413.9 | I20 I20.0 I20.1 I20.8 I20.9 |
| Hypertension | 401 401.0 401.1 401.9 | I10 I11 I11.0 I11.9 I12 I12.0 I12.9 I13 I13.0 I13.1 I13.2 I13.9 I15 I15.0 I15.1 I15.2 I15.8 I15.9 |
| Myocardial Infarction | 410 410.00 410.01 410.02 410.10 410.11 410.12 410.20 410.21 410.22 410.30 410.31 410.32 410.40 410.41 410.42 410.50 410.51 410.52 410.60 410.61 410.62 410.70 410.71 410.72 410.80 410.81 410.82 410.90 410.91 410.92 | I21 I21.0 I21.1 I21.2 I21.3 I21.4 I21.9 I22 I22.0 I22.1 I22.8 I22.9 |
| Heart failure | 428 428.0 428.1 428.20 428.21 428.9 | I50 I50.0 I50.1 I50.9 |
| Stroke | 434 434.01 434.11 434.91 | I61 I61.0 I61.1 I61.2 I61.3 I61.4 I61.5 I61.6 I61.8 I61.9 I62 I62.0 I62.1 I62.9 I63 I63.0 I63.1 I63.2 I63.3 I63.4 I63.5 I63.6 I63.8 I63.9 I64 |
| Diabetes | 250 250.00 250.01 250.02 250.03 250.10 250.11 250.12 250.13 250.20 250.21 250.22 250.23 250.30 250.31 250.32 250.33 250.40 250.41 250.42 250.43 250.50 250.51 250.52 250.53 250.60 250.61 250.62 250.63 250.70 250.71 250.72 250.73 250.81 250.82 250.83 250.90 250.91 250.92 250.93 | E10 E11 E12 E13 E14 |
| Stomach Ulcer | 531 531.00 531.01 531.10 531.11 531.20 531.21 531.30 531.31 531.40 531.41 531.50 531.51 531.60 531.61 531.70 531.71 531.90 531.91 532.00 532.01 532.10 532.11 532.20 532.21 532.30 532.31 532.40 532.41 532.50 532.51 532.60 532.61 532.70 532.71 532.90 532.91 533.00 533.01 533.10 533.11 533.20 533.21 533.30 533.31 533.40 533.41 533.50 533.51 533.60 533.61 533.70 533.71 533.90 533.91 | K25 K26 K27 |
| Depression | 296.20 296.21 296.22 296.23 296.24 296.25 296.26 296.30 296.31 296.32 296.33 296.34 296.35 296.36 | F32 F32.0 F32.1 F32.2 F32.3 F32.8 F32.9 F33 F33.0 F33.1 F33.2 F33.3 F33.4 F33.8 F33.9 F34 F34.0 F34.1 F34.8 F34.9 F38 F38.0 F38.1 F38.8 F39 |

*Table 2 - Comorbidities over time (once a patient reports a comorbidity, they are then coded as having this comorbidity for every subsequent follow-up they attended)*

|  | Follow-up (years) | | | | | | | |
| --- | --- | --- | --- | --- | --- | --- | --- | --- |
| N (% of total who attended follow-up) | 1 | 2 | 3 | 5 | 7 | 10 | 15 | 20 |
| Total patients who attended follow-up | 928 | 860 | 817 | 766 | 626 | 597 | 466 | 346 |
| Neoplasm (chapter II) | 12 (1.3) | 11 (1.3) | 18 (2.2) | 25 (3.3) | 41 (6.6) | 50 (8.4) | 62 (13.3) | 68 (19.7) |
| Diseases of blood and blood forming organs and certain immune mechanism disorders (chapter III) | 5 (0.5) | 12 (1.4) | 15 (1.8) | 18 (2.1) | 17 (2.7) | 21 (3.5) | 29 (6.2) | 21 (6.1) |
| Endocrine, nutritional and metabolic diseases (chapter IV) | 46 (5.0) | 49 (5.7) | 54 (6.6) | 63 (8.2) | 67 (10.7) | 73 (12.2) | 68 (14.6) | 66 (19.1) |
| Mental and behavioural disorders (chapter V) | 7 (0.8) | 14 (1.6) | 14 (1.7) | 24 (3.1) | 30 (4.8) | 40 (6.7) | 43 (9.2) | 44 (12.7) |
| Diseases of the nervous system (chapter VI) | 17 (1.8) | 18 (2.1) | 19 (2.3) | 27 (3.5) | 25 (4.0) | 46 (7.7) | 50 (10.7) | 51 (14.7) |
| Diseases of the circulatory system (chapter IX) | 62 (6.7) | 86 (10.0) | 103 (12.6) | 119 (15.5) | 130 (20.8) | 160 (26.8) | 136 (29.2) | 117 (33.8) |
| Diseases of the respiratory system (chapter X) | 52 (5.6) | 73 (8.5) | 87 (10.7) | 113 (13.3) | 160 (25.6) | 191 (32.0) | 198 (42.5) | 174 (50.3) |
| Diseases of the digestive system (chapter XI) | 35 (3.8) | 55 (6.4) | 62 (7.6) | 72 (9.4) | 79 (12.6) | 98 (16.4) | 118 (25.3) | 109 (31.5) |
| Diseases of the skin and subcutaneous tissues (chapter XII) | 18 (1.9) | 21 (2.4) | 24 (2.9) | 31 (4.1) | 36 (5.8) | 41 (6.9) | 71 (15.2) | 64 (18.5) |
| Diseases of the musculoskeletal system and connective tissue (chapter XIII) | 18 (1.9) | 32 (3.7) | 42 (5.1) | 59 (7.7) | 66 (10.5) | 98 (16.4) | 145 (31.1) | 138 (39.9) |
| Angina | 15 (1.6) | 21 (2.4) | 22 (2.7) | 24 (3.1) | 22 (3.5) | 54 (9.1) | 48 (10.3) | 42 (12.1) |
| Hypertension | 32 (3.5) | 43 (5.0) | 56 (6.9) | 61 (8.0) | 174 (27.8) | 234 (39.2) | 189 (40.6) | 143 (41.3) |
| Myocardial Infarction | 2 (0.2) | 6 (0.7) | 10 (1.2) | 11 (1.4) | 28 (4.5) | 38 (6.4) | 31 (6.7) | 24 (6.9) |
| Heart failure | 1 (0.1) | 1 (0.1) | 2 (0.2) | 5 (0.7) | 9 (1.4) | 22 (3.7) | 9 (1.9) | 5 (1.5) |
| Stroke | 0 | 0 | 0 | 2 (0.3) | 24 (3.8) | 28 (4.7) | 22 (4.7) | 14 (4.1) |
| Diabetes | 25 (2.7) | 27 (3.1) | 30 (3.7) | 31 (4.1) | 43 (6.9) | 46 (7.7) | 35 (7.5) | 32 (9.3) |
| Stomach Ulcer | 2 (0.2) | 4 (0.5) | 7 (0.9) | 9 (1.2) | 54 (8.6) | 70 (11.7) | 62 (13.3) | 46 (13.3) |
| Depression | 1 (0.1) | 3 (0.4) | 5 (0.6) | 13 (1.7) | 141 (22.5) | 200 (33.5) | 166 (35.6) | 135 (39.0) |
