# Supplementary material for: Twenty‐Year Outcome and Association Between Early Treatment and Mortality and Disability in an Inception Cohort of Patients With Rheumatoid Arthritis: Results From the Norfolk Arthritis Register
Source: Arthritis Rheumatol. 2017 Jul 10;69(8):1566–75. doi: 10.1002/art.40090 (PMC5600136; doi:10.1002/art.40090)
Supplement: Supplementary file 2 [file ART-69-1566-s002.docx]

**Supplementary file 2**

*Inverse probability of treatment / censoring weights*

Confounding by indication occurs in observational studies when patients who have more severe disease are treated differently from patients with less severe disease (1). Patients who received early treatment are likely to have more severe disease than patients treated later. Furthermore patients who left the study early are likely to differ from patients who remained in the study, biasing the results. Propensity score modelling can be used to balance the factors that predict initiation of first treatment. However clinicians make many subsequent treatment decisions over the course of the disease progression of patients and the factors that may prompt physicians to initiate / terminate treatment are also on the proposed causal pathway between early treatment and reduced severity of long-term outcomes. Therefore simply including these variables as covariates in the models would underestimate the association between early treatment and long-term outcomes, as these path variables are effectively held constant (2). Therefore inverse probability of treatment (IPTW) / censoring weights (IPCW) were used to weight the regression analyses.

To calculate the IPTW, each subject’s probability of having their own treatment history was estimated. This is because each of the treatment groups is likely to have different treatment histories, based on factors that both predict time-varying treatment and future outcome. For instance, disease activity (e.g. swollen joint count) at a given time-point is associated with future Health Assessment Questionnaire (HAQ) score, the initiation/termination of future treatment and is itself influenced by previous treatment (3). Thus if we weigh the analysis based on the probability of a patient receiving their own treatment history given their set of prognostic factors, this will create a pseudo-population in which the time-varying treatment histories of patients are unrelated to their time-varying confounders. This allows for an analysis of the association between early treatment and long-term outcomes, unbiased by the different disease severities between the groups.

Patients in NOAR were seen at multiple time-points. A single dichotomous variable was created that denoted whether a patient was receiving treatment (synthetic disease modifying anti-rheumatic drugs or steroids) at each time-point. A logistic regression model with the dependent variable being this treatment variable and with baseline, current assessment and previous assessment variables as covariates (table 1) was used to estimate the probability that patients were on treatment at each assessment. As an estimate of the probability of the patients’ actual treatment histories was required, rather than the probability that patients were on treatment at each assessment, the probabilities were subtracted from one on assessments where patients were not receiving treatment. To account for the complete treatment history of the patients up to each assessment the cumulative probability of treatment was calculated. This was done by multiplying the probability of receiving the treatment that the patient received at each assessment by all the probabilities from the prior assessments. Thus the cumulative probability of a patient receiving their own treatment history up to assessment two would be the probability that the patient received the treatment that they received at baseline multiplied by the probability at assessment one multiplied by the probability at assessment two. These values can, informally, be thought of as a patient’s conditional probability of receiving the treatment history that they actually received, given their past treatment history and clinical features (4). To create the inverse probability weight, the reciprocal of this cumulative probability at each assessment is taken (i.e. 1 / cumulative probability of each patient receiving their actual treatment history up to the current assessment).

However these weights are often highly variable and fail to approximate a normal distribution. Therefore these weights were stabilised. This was achieved by repeating the process above, except only using baseline variables in the logistic regression to calculate the probability that a patient received treatment at a given assessment. This results in two cumulative probabilities, one that results from a logistic regression conditioned only on baseline variables (the numerator) and one that results from a logistic regression conditioned on baseline and time-varying variables (the denominator). The stabilised inverse probability of treatment weight is then derived by dividing the numerator by the denominator.

To calculate stabilised IPCW, the same procedure as above was used. Instead of predicting whether patients were on treatment at each assessment, the probability that a patient was censored by the next assessment was estimated. Finally the IPTW and the IPCW were multiplied together to give a final inverse weight. These weights were then used to weight the covariates in the regression models. In the weighted analyses, the distribution of the time-varying confounders is the same across the treatment groups (5). This allows an unbiased estimate of the association between delays in initiating treatment and long-term outcome.

*Table 1 – Variables used in inverse probability of treatment / censor weights*

| Variable | Variable type |
| --- | --- |
| *Demographic* |  |
| Age at onset | Continuous (years) |
| Gender | Dichotomous (male / female) |
| Time between symptom onset and follow-up | Continuous (months) |
| Baseline smoking status | Ordinal (never / former / current) |
| *Serology* |  |
| ACPA | Dichotomous (positive / negative) |
| RF | Dichotomous (positive / negative) |
| Baseline CRP | Continuous (mg/l) |
| *Disease activity and severity* |  |
| Baseline DAS28 | Continuous |
| Swollen joint (51) count at baseline, current assessment and previous assessment | Continuous |
| Tender joint (51) count at baseline, current assessment and previous assessment | Continuous |
| HAQ score at baseline, current assessment and previous assessment | Continuous |
| 2010 RA criteria at baseline | Dichotomous (met / mid not meet criteria) |
| *Comorbidity* |  |
| Variables indicating specific ICD9 / 10 chapters as well as specific comorbidities (detailed in methods and in supplementary file 1) | Dichotomous (does / does not have comorbidity) |
| Treatment |  |
| On DMARDs at baseline and previous assessment | Dichotomous (taking / not taking DMARDs) |

*ACPA = anti-citrullinated protein antibodies, CRP = C-reactive protein, DAS28 = Disease activity score, DMARD = disease modifying anti-rheumatic drugs, HAQ = Health Assessment Questionnaire, ICD = International Classification of Diseases, l = litres, mg = milligrams, RA = rheumatoid arthritis, RF = rheumatoid factor*

Reference List

(1) Psaty BM, Siscovick DS. Minimizing bias due to confounding by indication in comparative effectiveness research: the importance of restriction. JAMA 2010; 304:897-8.

(2) Choi HK, Hernan MA, Seeger JD, Robins JM, Wolfe F. Methotrexate and mortality in patients with rheumatoid arthritis: a prospective study. Lancet 2002; 359:1173-7.

(3) Robins JM, Hernan MA, Brumback B. Marginal structural models and causal inference in epidemiology. Epidemiology 2000; 11:550-60.

(4) Fewell Z, Hernán M, Wolfe F, Tilling K, Choi HK, Sterne J. Controlling for time-dependent confounding using mardinal structural models. The Stata Journal 2004; 4:402-20.

(5) Farragher TM, Lunt M, Fu B, Bunn D, Symmons DP. Early treatment with, and time receiving, first disease-modifying antirheumatic drug predicts long-term function in patients with inflammatory polyarthritis. Ann Rheum Dis 2010; 69:689-95.
