# Supplementary material for: Twenty‐Year Outcome and Association Between Early Treatment and Mortality and Disability in an Inception Cohort of Patients With Rheumatoid Arthritis: Results From the Norfolk Arthritis Register
Source: Arthritis Rheumatol. 2017 Jul 10;69(8):1566–75. doi: 10.1002/art.40090 (PMC5600136; doi:10.1002/art.40090)
Supplement: Supplementary file 3 — – Baseline and follow‐up characteristics of the total IP population [file ART-69-1566-s003.docx]

|  | Cohort (N=1000) | ET (N=211) § | LT (N=347) § | NT (N=442) § | |  |
| --- | --- | --- | --- | --- | --- | --- |
|  | Median (IQR) [% complete data] | Median (IQR) [% complete data] | Median (IQR) [% complete data] | Median (IQR) [% complete data] | | p* |
| Age at onset, (years) | 54 (41, 66)  [100] | 62 (48, 71)  [100] | 53 (42, 64)  [100] | 52 (39, 65)  [100] | | 0.0001~ |
| Gender, N(%) female | 650 (65)  [100] | 113 (53.6)  [100] | 242 (69.7)  [100] | 295 (66.7)  [100] | | <0.001^ |
| Symptom duration, months | 5.1 (2.7, 9.4)  [100] | 3.4 (2.1, 5.4)  [100] | 6.9 (3.8, 11.8)  [100] | 4.9 (2.3, 9.3)  [100] | | 0.0001~ |
| Swollen joint count 28 | 5 (1, 11)  [100] | 8 (3, 15)  [100] | 6 (2, 12)  [100] | 3 (1, 7)  [100] | | 0.0001~ |
| Swollen joint count 51 | 6 (2, 13)  [100] | 9 (4, 17)  [100] | 8 (3, 15)  [100] | 4 (1, 9)  [100] | | 0.0001~ |
| Tender joint count 28 | 5 (2, 12)  [100] | 7 (2, 15)  [100] | 6 (2, 12)  [100] | 4 (1, 9)  [100] | | 0.0001~ |
| Tender joint count 51 | 7 (3, 16)  [100] | 10 (4, 20)  [100] | 9 (3, 18)  [100] | 6 (2, 13)  [100] | | 0.0001~ |
| CRP, mg/l | 5 (0, 16)  [79.8] | 13 (5, 37.2)  [83.9] | 7 (1, 17)  [80.0] | 2 (0, 8)  [78.5] | | 0.0001~ |
| DAS28 | 3.9 (2.9, 5.0)  [79.8] | 4.7 (3.7, 5.6)  [83.9] | 4.2 (3.3, 5.1)  [80.0] | 3.3 (2.4, 4.4)  [78.5] | | 0.0001~ |
| HAQ | 0.75  (0.25, 1.38)  [98.8] | 1.25  (0.50, 1.75)  [98.6] | 0.88  (0.38, 1.50)  [98.3] | 0.50  (0.13, 1.00)  [99.3] | | 0.0001~ |
| Smoking status:  Never, N(%)  Ex, N(%)  Current, N(%) | 317 (31.7)  412 (41.2)  270 (27.0)  [99.9] | 61 (29.1)  96 (45.7)  53 (25.2)  [99.5] | 111 (32.0)  131 (37.8)  105 (30.3)  [100] | 145 (32.8)  185 (41.9)  112 (25.3)  [100] | | 0.310^ |
| RF status:  Positive, N(%)  Negative, N(%) | 248 (28.5)  622 (71.5)  [87.0] | 74 (38.1)  120 (61.9)  [91.9] | 119 (39.9)  179 (60.1)  [85.9] | 55 (14.6)  323 (92.6)  [85.5] | | <0.001^ |
| Anti-CCP status:  Positive, N(%)  Negative, N(%) | 214 (27.5)  565 (72.5)  [77.9] | 73 (42.2)  100 (57.8)  [82.0] | 142 (55.3)  115 (44.8)  [74.1] | 26 (7.5)  323 (92.6)  [79.0] | | <0.001^ |
| Current sDMARD use, N(%) | 138 (13.8)  [100] | 95 (45.0)  [100] | 43 (12.4)  [100] | 0 (0.0)  [100] | | <0.001^ |
| Time to first treatment, (months) | 9.0 (4.0, 20.2) [100] | 3.0 (1.9, 4.5) [100] | 17.0 (10.0, 44.9)  [100] | | - |  |

**Supplementary file 3 – Baseline and follow-up characteristics of the total IP population**

*Table 1 – Baseline characteristics of the cohort of patients with RA*

** p values resulting from comparison of baseline score across treatment groups, ~ = Kruskal-Wallis, ^ = χ^2^,*

*§Early treatment (ET) = treatment ≤ 6 months after symptom onset, late treatment (LT) = treatment > 6 months after symptom onset, Never treatment (NT) = patient never received DAMRDs or steroids during follow-up.*

*Anti-CCP = Anti-citrullinated peptide antibodies, DAS28 = Disease activity score (28), HAQ = Health assessment questionnaire, IQR = Interquartile range, l = Litres, mg = Milligrams, N = Number of patients, RA = Rheumatoid arthritis, RF = Rheumatoid factor, sDMARD = Synthetic Disease Modifying Anti-Rheumatic Drugs*

*Table 2 – Median 51 SJC/TJC and HAQ scores over follow-up for the whole IP cohort and stratified by treatment group (N=1000)*

|  | Follow, up year score, median (IQR) | | | | | | | | |
| --- | --- | --- | --- | --- | --- | --- | --- | --- | --- |
|  | 0 | 1 | 2 | 3 | 5 | 7 | 10 | 15 | 20 |
| SJC 51 |  |  |  |  |  |  |  |  |  |
| Total | 6 (2, 13) | 2 (0, 7) | 2 (0, 6) | 2 (0, 5) | - | - | 1 (0, 4) | 1 (0, 3) | 0 (0, 2) |
| ET | 9 (4, 17) | 3 (0, 9) | 2 (0, 7) | 2 (0, 7) | - | - | 2 (0, 5.5) | 1 (0, 5) | 0 (0, 2) |
| LT | 8 (3, 15) | 3 (1, 9) | 3 (0, 8) | 2 (0, 7) | - | - | 2 (0, 5) | 1 (0, 4) | 1 (0, 3) |
| NT | 4 (1, 9) | 1 (0, 4) | 1 (0, 3) | 1 (0, 3) | - | - | 0 (0, 3) | 0 (0, 2) | 0 (0, 2) |
| TJC |  |  |  |  |  |  |  |  |  |
| Total | 7 (3, 16) | 4 (1, 11) | 4 (1, 10.5) | 3 (1, 10) | - | - | 2 (0, 11) | 3 (0, 10) | 2 (0, 10) |
| ET | 10 (4, 20) | 4 (1, 14) | 3 (1, 12) | 3.5 (0, 13) | - | - | 2 (1, 11) | 2 (0, 10) | 2 (0, 5) |
| LT | 9 (3, 18) | 6 (2, 14) | 6 (2, 15) | 4 (1, 13) | - | - | 4 (1, 14) | 4 (0, 14) | 4 (0, 13) |
| NT | 6 (2, 13) | 3 (1, 8) | 3 (0, 6) | 2 (0, 6) | - | - | 2 (0, 6) | 2 (0, 7) | 2 (0, 6) |
| HAQ |  |  |  |  |  |  |  |  |  |
| Total | 0.75  (0.25, 1.38) | 0.50  (0.00, 1.25) | 0.50  (0.00, 1.25) | 0.63  (0.00, 1.38) | 0.75  (0.13, 1.50) | 0.75  (0.25, 1.63) | 0.88  (0.25, 1.69) | 1.00  (0.25, 1.75) | 1.00  (0.25, 1.88) |
| ET | 1.25  (0.5, 1.75) | 0.75  (0.13, 1.44) | 0.75  (0.13, 1.63) | 0.88  (0.25, 1.63) | 1.00  (0.25, 1.88) | 1.13  (0.38, 1.75) | 1.25  (0.25, 2.13) | 1.38  (0.63, 2.00) | 1.38  (0.75, 2.00) |
| LT | 0.88  (0.38, 1.50) | 0.75  (0.25, 1.50) | 0.88  (0.25, 1.63) | 0.88  (0.25, 1.63) | 1.00  (0.38, 1.75) | 1.00  (0.38, 1.75) | 1.25  (0.50, 1.88) | 1.19  (0.50, 1.88) | 1.25  (0.38, 2.00) |
| NT | 0.50  (0.13, 1.00) | 0.25  (0.00, 0.88) | 0.38  (0.00, 0.75) | 0.25  (0.00, 0.88) | 0.38  (0.00, 1.06) | 0.50  (0.13, 1.13) | 0.50  (0.13, 1.13) | 0.63  (0.00, 1.38) | 0.50  (0.13, 1.50) |
| sDMARD |  |  |  |  |  |  |  |  |  |
| Total | 138 (13.8) | 264 (28.5) | 260 (30.2) | 259 (31.7) | 232 (30.3) | 204 (32.6) | 195 (32.7) | 171 (36.7) | 134 (38.7) |
| ET | 95 (45.0) | 122 (59.5) | 113 (59.5) | 103 (56.0) | 86 (52.8) | 70 (55.1) | 69 (58.0) | 57 (60.6) | 40 (59.7) |
| LT | 43 (12.4) | 142 (41.4) | 147 (44.3) | 156 (49.2) | 146 (48.5) | 134 (50.2) | 126 (49.6) | 114 (55.1) | 94 (58.8) |
| NT  *ET = early treatment, HAQ = Health Assessment Questionnaire, IQR = Interquartile range, LT = late treatment, N = Number, NT = never treatment, sDMARD = synthetic disease modifying anti-rheumatic drug, SJC = swollen joint count, TJC = tender joint count*  *§ = N(%) displayed for current sDMARD use* | 0 (0.0) | 0 (0.0) | 0 (0.0) | 0 (0.0) | 0 (0.0) | 0 (0.0) | 0 (0.0) | 0 (0.0) | 0 (0.0) |
