# Supplementary material for: Twenty‐Year Outcome and Association Between Early Treatment and Mortality and Disability in an Inception Cohort of Patients With Rheumatoid Arthritis: Results From the Norfolk Arthritis Register
Source: Arthritis Rheumatol. 2017 Jul 10;69(8):1566–75. doi: 10.1002/art.40090 (PMC5600136; doi:10.1002/art.40090)
Supplement: Supplementary file 4 — – attrition from the cohort over 20 years [file ART-69-1566-s004.docx]

**Supplementary file 4 – attrition from the cohort over 20 years**

The number of patients at baseline was 1000. Over the course of 20 years 651 (65.1%) patients were lost to follow-up. This was made up of 250 men (71.4% of baseline number of men) and 401 women (61.7% of baseline number of women). Figure 1 shows that the rate of attrition was relatively constant.

Figure 2 shows the proportion of patients leaving for the three main reasons (death, declining further follow-up, patient being lost to research team). Each bar represents the total number of patients lost to follow-up for these three reasons between two follow-ups. The main reason patients left the study during the first three years of the study was declining further follow-up visits. From five years and beyond, death was the main reason patients left the study. As time went on patients grew older, thus more patients died in the latter years of the study.

Figures 3 and 4 show the proportions of patients leaving the study for the three main reasons, stratified by gender. After the first year, the main reason men left the study was due to death (figure 3). For women, the main reason that patients left the study fluctuated over time, changing between death and patients declining follow-up.

*
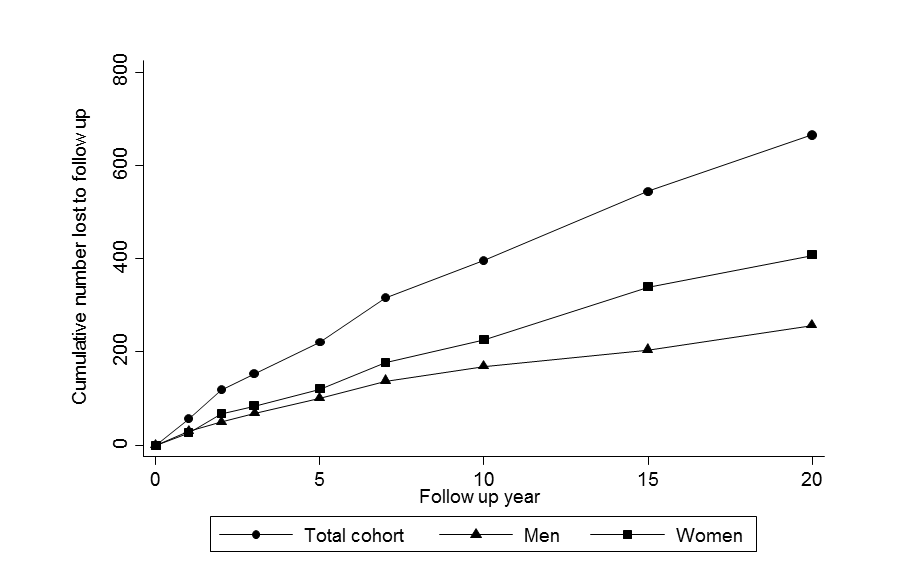
*

*Figure 1 – cumulative number of patients lost to follow-up over time*

*
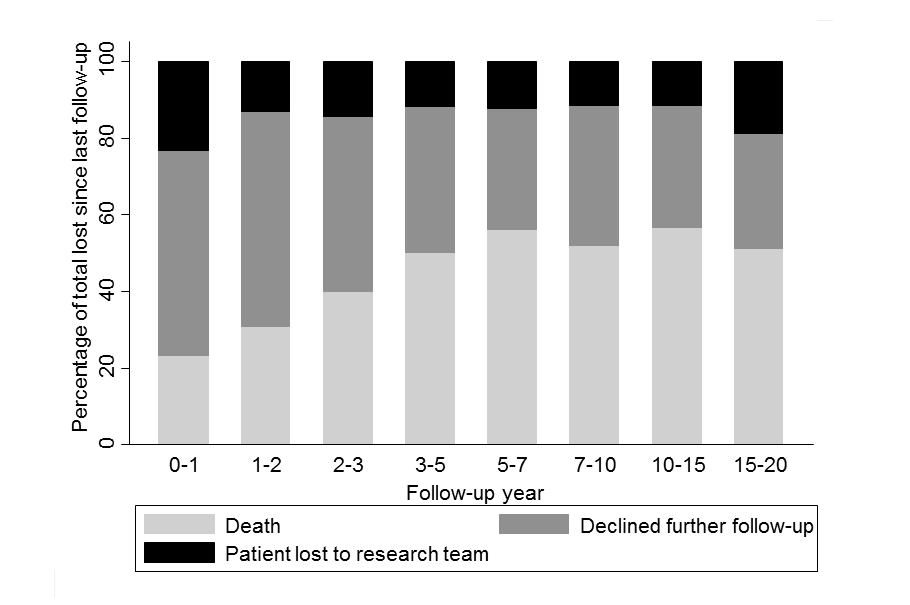
*

*Figure 2 – proportions of the total number of patients leaving the study for different reasons*


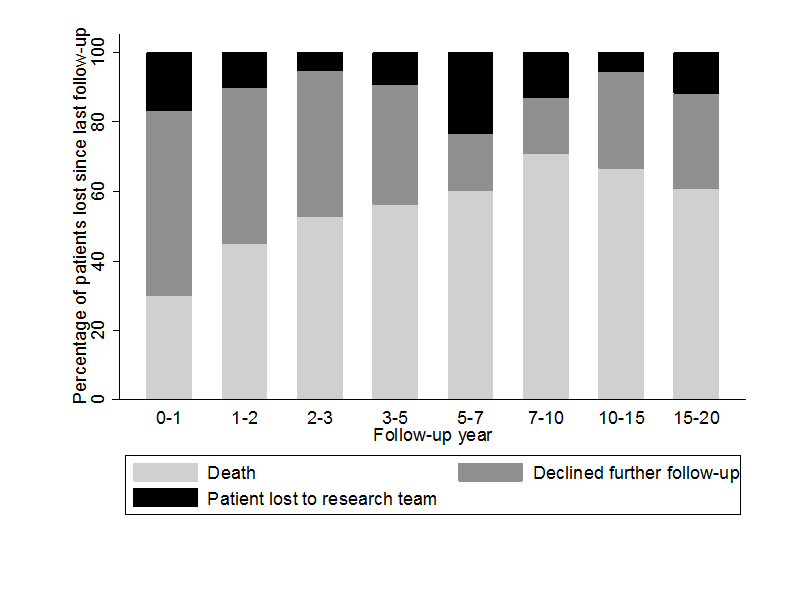


*Figure 3 – Proportions of the total number of men leaving the study for different reasons*


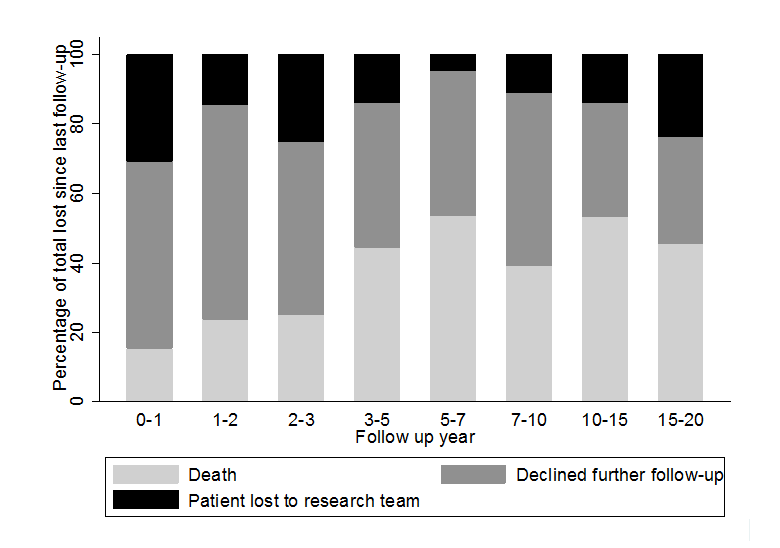


*Figure 4 – Proportions of the total number of women leaving the study for different reasons*
